# Bilateral Medial Epicondyle Fracture Without Elbow Dislocation in a High-Level Adolescent Gymnast Athlete: A Rare Case

**DOI:** 10.7759/cureus.33105

**Published:** 2022-12-29

**Authors:** Anthimos Keskinis, Konstantinos G Makiev, Paraskevas Georgoulas, Efthymios Iliopoulos, Athanasios N Ververidis

**Affiliations:** 1 Sports Injuries Unit, Department of Orthopaedics, University General Hospital of Alexandroupolis, Democritus University of Thrace, Alexandroupolis, GRC

**Keywords:** gymnast athlete, sport injury, case report, elbow dislocation, adolescent, bilateral medial epicondyle fracture

## Abstract

Isolated medial epicondyle fracture constitutes a common fracture in children’s and adolescent’s elbow and is highly associated with an elbow dislocation. Cases with bilateral medial epicondyle fracture with concomitant elbow dislocation have been previously described in literature, while cases without an associated elbow dislocation are lacking. In this article, a bilateral medial epicondyle fracture without elbow dislocation in an adolescent high-level gymnast athlete is reported. To the best of the authors’ knowledge, this is the first case report in literature regarding such an extremely rare traumatic elbow injury.

## Introduction

Isolated medial epicondyle fracture constitutes one of the most common fractures in children’s and adolescents’ elbows. It is associated with an elbow dislocation in approximately 50% of the patients [1]. Cases with bilateral medial epicondyle fracture and elbow dislocation have also been described [2,3]. However, bilateral medial epicondyle fracture without an associated elbow dislocation is extremely rare and has never been reported in literature [4].

The treatment of such injuries has not yet standardized. Thus, each patient should be treated individually according to the existence of major or minor surgical indications, the functional requirements of the patient and the surgeon’s preference and experience [1,2,4,5].

In this case report, a bilateral medial epicondyle fracture without elbow dislocation in an adolescent high-level gymnast athlete is presented. To the best of the authors’ knowledge, this is the first case report in literature regarding such an extremely rare traumatic elbow injury.

## Case presentation

A 15-year-old male competitive gymnast athlete, with right dominant hand, presented to the emergency department with a bilateral elbow traumatic injury while performing in a competition. According to personal history, the patient’s handgrip slipped from the gymnastics horizontal bar during the execution of a backward giant circle on a high bar. At the time of landing, the patient’s axis was transverse to the floor with the head upside down. The shoulders were also flexed in 180° and the elbows in 90°. The patient’s weight was axially loaded on both flexed elbows.

**Figure 1:**
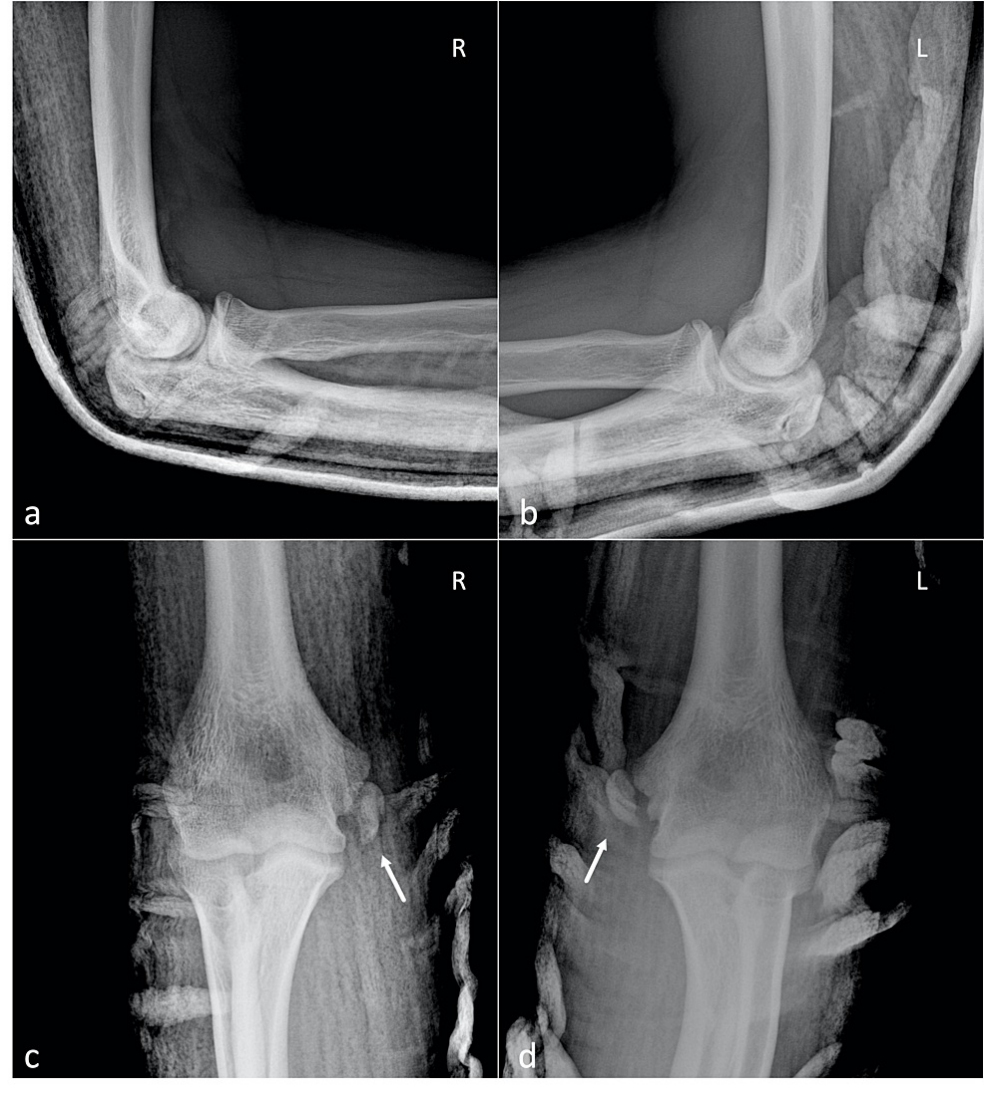
Pre-operative radiographs of both elbows Pre-operative lateral (a, b) and anteroposterior (c, d) views of both elbows showing the bilateral medial epicondyle fractures (white arrows). L: Left; R: Right

Focused clinical examination revealed significant swelling and bruising of both elbows with painful and restricted range of motion (ROM) in both flexion and extension. Tenderness during palpation was localized at the medial epicondyles while the medial ligamentous complex was unstable in the valgus stress test. There was no neurovascular injury. No other injuries were identified. The Beighton score was 0/9 points. Standard elbow radiographs (antero-posterior and lateral views) established the diagnosis of bilateral medial humeral epicondyle fracture (Figure 1) and, in combination with the clinical examination, excluded the traumatic dislocation of both elbows.

**Figure 2:**
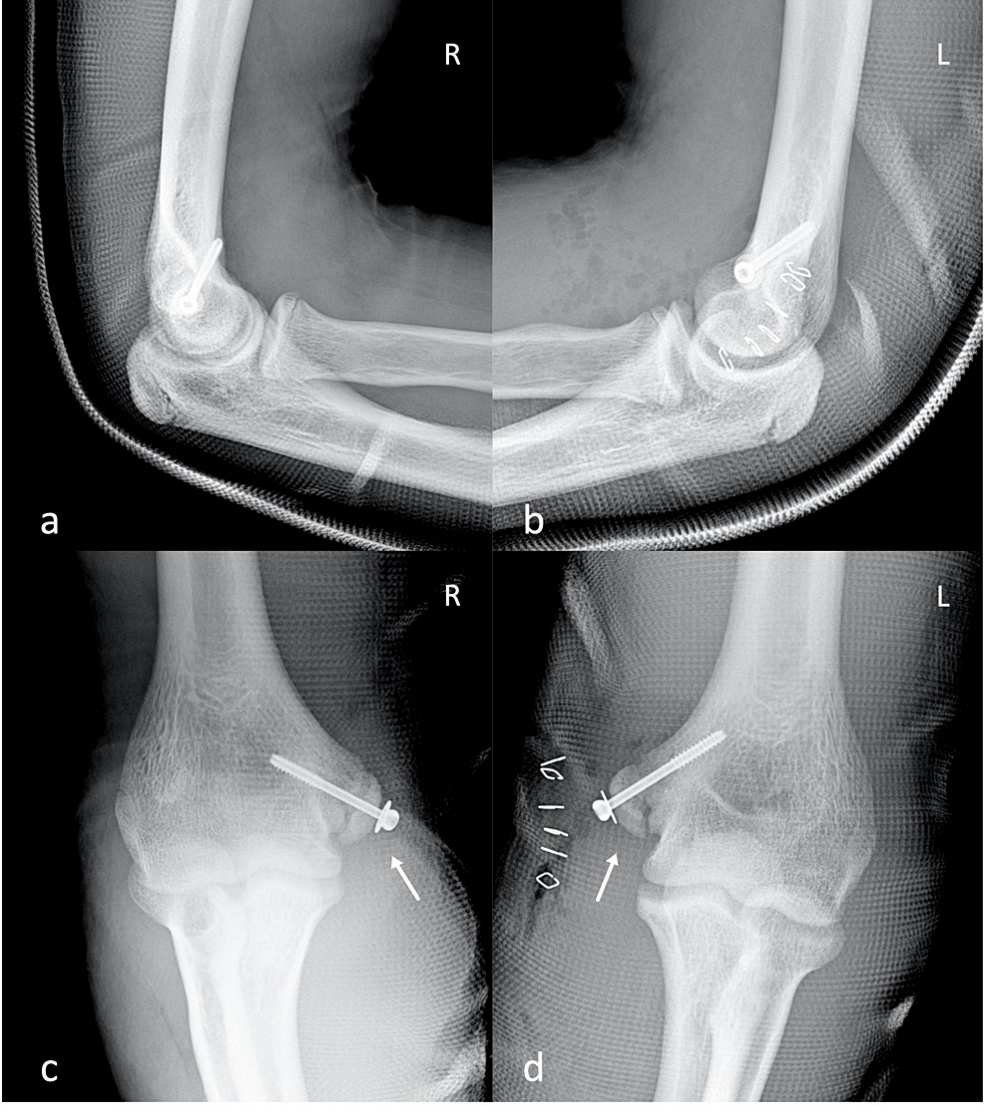
Post-operative radiographs of both elbows Post-operative lateral (a, b) and anteroposterior (c, d) views of both elbows showing the anatomic fixation of the fractures with a screw (white arrows). L: Left; R: Right

Surgical treatment was immediately planned under general anesthesia. The patient was placed in supine position and both hands were operated by the same senior surgeon (Dr. ANV). The ulnar nerve was carefully dissected and released through a small posteromedial incision without soft tissue or “bony” anterior ulnar nerve transposition. The medial epicondyles of both humeri were then exposed and internally fixated using one cannulated partially threaded cancellous screw with a washer (Figure 2). Stress radiographs were performed prior to the wound closure to confirm the stability of both elbows

**Figure 3:**
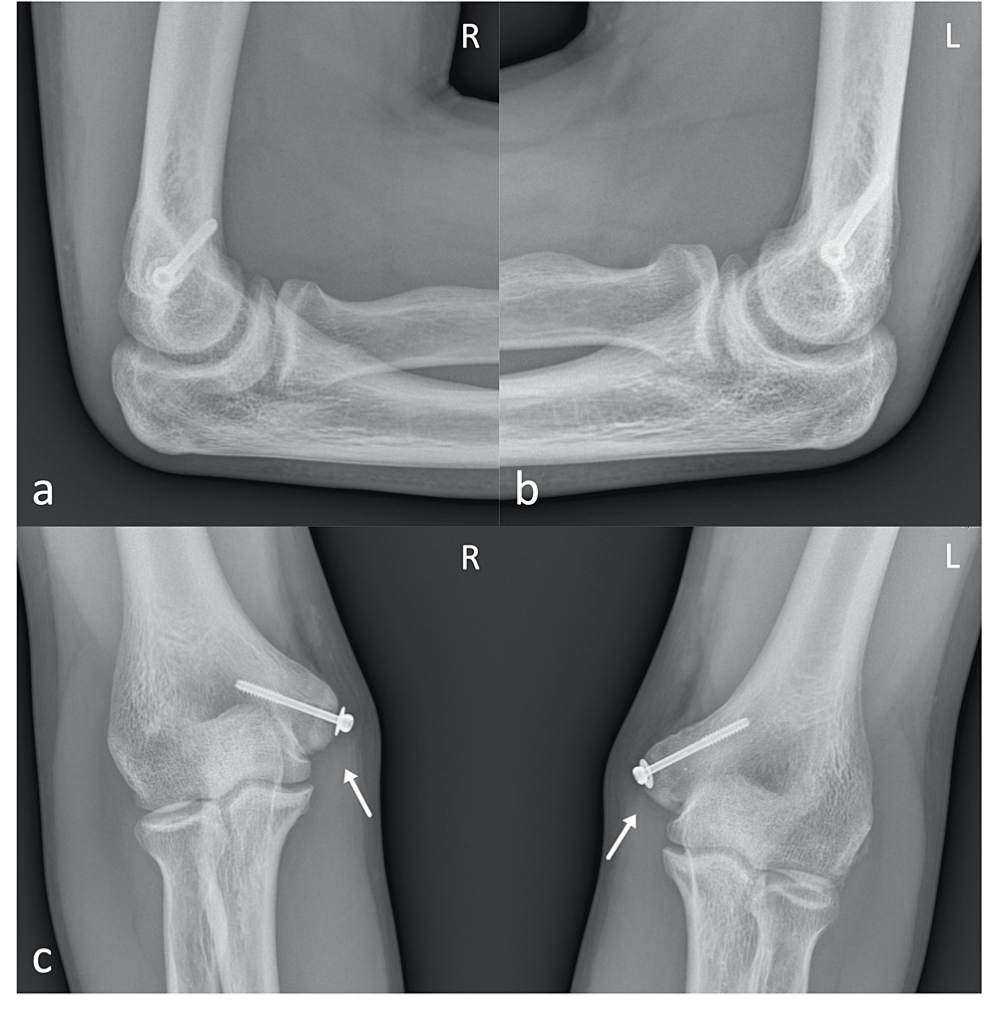
Radiographs, 4 months post-operatively, showing the healed fractures Lateral (a, b) and anteroposterior (c, d) views of both elbows four months post operation, showing the healed fractures (white arrows). L: Left; R: Right

**Figure 4:**
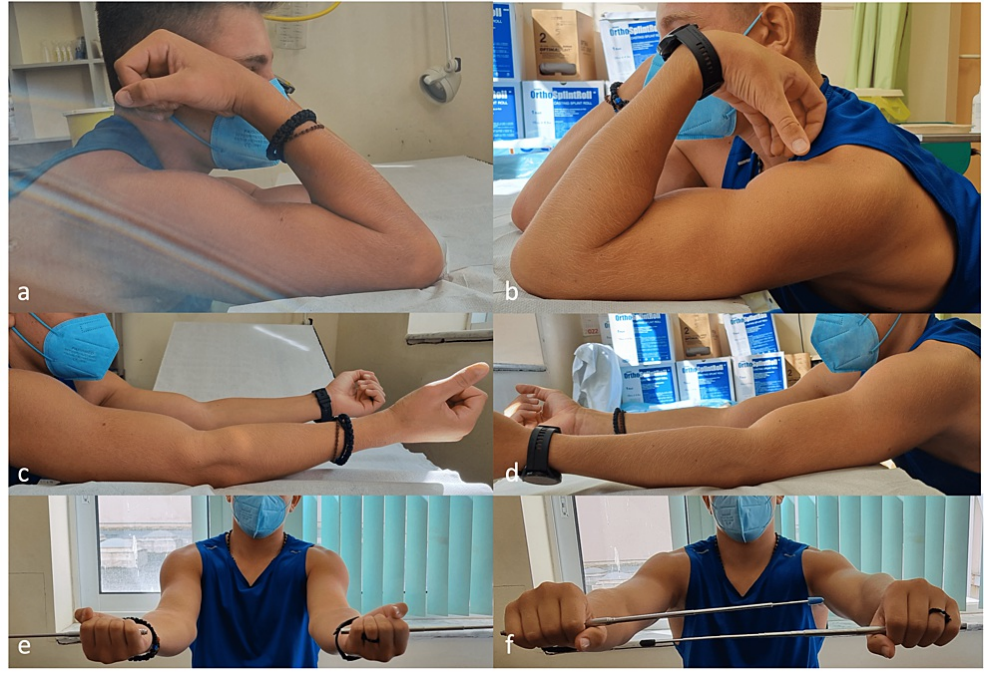
Flexion, extension, supination, and pronation of both elbow joints

A posterior splint was applied for the first two weeks following surgery with both elbows in 90° of flexion and neutral supination/pronation. At the two-week follow-up, the sutures were removed and splints were switched to hinged elbow braces in order to initiate active and passive motion as tolerated but simultaneously protect the elbows from varus/valgus stresses. Four weeks following surgery, the hinged braces were removed, and the patient followed a standard physiotherapy protocol for further four weeks. During the last follow-up period of four months, the patient did not have any ulnar nerve symptoms and the fracture sites had been healed (Figure 3). The ROM of the right elbow was between 20° of extension to 140° of flexion, while on the left from 15° of extension to 135° of flexion. Both supination and pronation were fully restored (180°) (Figure 4). The patient was satisfied with the functional outcome and was encouraged to gradually come back to his preinjury activities.

## Discussion

This case concerns a high-level adolescent athlete with bilateral gross elbow instability due to medial epicondyle fractures after an injury. These high demand patient’s elbows were daily exposed under high-level of stress during sporting activities, prior to trauma, therefore a maximum long-term stability was required. These factors constitute three out of four of the relative indications for surgical treatment of medial epicondyle fracture in adolescents [6]. According to Gottschalk et al., the relative factors for surgical indication are the ulnar nerve dysfunction, the valgus instability, the fracture displacement more than 2 mm, and the high-demand patients [6]. Surgical treatment with open reduction and internal fixation decreases the non-union frequency and averts valgus instability by tension restoration of the medial collateral ligament [4].

Controversies regarding the conservative treatment do exist in the literature. Non operative treatment has been proposed for minimally displaced fragments (< 5 mm) in low-demand patients who are not competing at a high level [1]. The most common complication is radiographic nonunion or valgus instability in more than 60% of the patients [5]. The paradox of nonunion in low-demand patients is that the long-term functional results are good enough for them [4]. The residual valgus instability of the elbow is attributed to the medial collateral ligament laxity and functional lengthening due to the non-anatomical healing (malunion) of the medial epicondyle [2,5].

In this case, the epiphyseal plates of the 15-year-old patient were closed and the articular surface was intact due to the extra-articular localization of the medial epicondyle in adolescents [4]. The displaced medial epicondyle fracture of both elbows was localized above the articular surface of the distal humerus. Based on this fact, it was considered that the fragment was not involving the joint surface. Thus, a CT scan was not indicated [1].

When the mechanism of injury includes a direct elbow trauma in combination with an avulsion mechanism involving indirect muscular pull, the injury is associated with elbow dislocation [6]. On the contrary, the mechanism of injury in our case was an isolated bilateral direct trauma, a fact that justifies the absence of elbow dislocation. To our knowledge, there is no other report in the literature describing a bilateral medial epicondyle fracture without an elbow dislocation [4]. However, similar injuries with concomitant elbow dislocation have been previously described [2,3,7].

The anatomically fixated medial epicondyle is the key for elbow stability because it constitutes the traction apophysis of all soft tissue medial stabilizers [5]. The medial collateral ligament of both elbows were intact and attached to the fragmented medial epicondyle. This phenomenon was confirmed by Park et al. in a retrospective study including 31 medial epicondyle fractures in adolescents without elbow dislocation [4]. None of these patients had a medial collateral ligament tear [4].

The preferable method for medial epicondyle fixation in skeletally mature adolescents is screw fixation, while in younger immature patients, Kirschner wires are indicated [5]. The benefits of screw fixation in comparison with Kirschner wires or conservative treatment are remarkable. The elbow is rapidly mobilized resulting in faster ROM recovery and sooner return to sports with high union rates [2]. At the same time, the risk of valgus instability is minimized and the symptomatic nonunion is prevented [5].

The screw washer is inserted according to the surgeon's preference. It increases the surface area for compression reducing the risk of intraoperative medial epicondyle fragmentation and screw penetration [1,2]. Approximately 60% of these patients develop hardware irritation and ask for screw removal in contrast with 0% of those without a washer [2].

The rehabilitation program depends on the preferred treatment method. When conservative treatment is chosen, a posterior splint with the elbow in 90° is applied for the first four weeks, followed by physiotherapy, to regain the normal ROM of the elbow. In contrary, when the fracture is internally fixated and maximum stability is achieved, the posterior splint can be changed to a hinged brace in just two weeks or even earlier [8-10]. Using the brace, the active/passive flexion and extension of the joint is encouraged, while simultaneously the elbow is protected from valgus strain, thus promoting earlier but safe mobilization, minimizing the chance of elbow stiffness [11,12]. According to a recent systematic review, both conservative and operative treatment can achieve satisfactory functional outcomes. Nevertheless, operatively treated patients start mobilizing their elbow significantly earlier, which consecutively results in a shorter time until returning to sport (average: 3.8 months) [12].

## Conclusions

In conclusion, this is the first report in literature describing an adolescent athlete with bilateral medial epicondyle fracture without an elbow dislocation. The treatment of both elbows was successfully done with anatomical internal fixation using a screw and a washer. The final clinical and functional outcomes were satisfactory, and the patient was able to return to his preinjury activities. This case report has a principal goal to widen the clinical and operative spectrum of orthopedic surgeons, especially for the management of medial epicondyle fracture in adolescent patients.
